# Case Report: Prolonged response to cabozantinib and pembrolizumab in treatment-refractory metastatic pancreatic ductal adenocarcinoma

**DOI:** 10.3389/fonc.2026.1675133

**Published:** 2026-01-26

**Authors:** Anna M. Reagan, Hannah G. McDonald, Jaewon Kang, Hoda Saghaeiannejad Esfahani, Caroline G. Wright, Charles J. Bailey, Carleton S. Ellis, Kaylyn R. Collette, Reema A. Patel, Mayte Murillo, Samantha F. Stokley, Michael J. Cavnar, Prakash K. Pandalai, Mautin T. Barry-Hundeyin, Jessica J. Moss, Joseph Kim

**Affiliations:** 1 Department of Surgery, University of Kentucky, Lexington, KY, United States; 2 Markey Cancer Center, University of Kentucky, Lexington, KY, United States; 3 University of Kentucky School of Medicine, Lexington, KY, United States; 4 Department of Toxicology and Cancer Biology, University of Kentucky, Lexington, KY, United States; 5 College of Pharmacy, University of Kentucky, Lexington, KY, United States; 6 Department of Internal Medicine, University of Kentucky, Lexington, KY, United States

**Keywords:** cabozantinib, case report, osteoclast-like giant cells, pancreatic cancer, pembrolizumab

## Abstract

Immunotherapy has yet to demonstrate durable efficacy in pancreatic ductal adenocarcinoma (PDAC) despite the fact that pancreatic cancer cells have higher levels of immune checkpoint expression than other cancer cell types in which immunotherapy efficacy has been well established. We observed effective cytotoxicity with the combination therapy of pembrolizumab (an anti-PD-1 monoclonal antibody, mAb) and cabozantinib (an anti-MET small molecule) *in vivo*. This served as the basis for an investigator-initiated phase II clinical trial (NCT05052723). The trial was open to patients with treatment-refractory metastatic PDAC with a single-arm treatment protocol of pembrolizumab and cabozantinib. Here, we report the case of a patient with metastatic PDAC who had received two prior lines of systemic therapy and was enrolled in our trial after presenting with pulmonary metastasis. She had a durable response to cabozantinib and pembrolizumab and remained on the trial for 25 months before demonstrating radiographic signs of disease progression. Notably, her initial pathology revealed poorly differentiated adenocarcinoma with features of undifferentiated carcinoma with osteoclast-like giant cells (UCOGC), which is an established rare variant of PDAC. Subsequent biopsies at metastatic sites revealed adenocarcinoma consistent with a pancreatic primary tumor. We hypothesize that the immune features of UCOGC, including increased PD-1 and PD-L1 expression and tumor-infiltrating lymphocytes, may have contributed to the effectiveness of this combination immunotherapy regimen in this patient’s response.

## Introduction

For many cancers (*e.g.*, colon cancer and melanoma), immunotherapy has produced groundbreaking improvements in survival (1, 2). Landmark trials have validated the approach of using an individual’s own immune system to enhance the therapeutic killing of cancer cells. However, in pancreatic ductal adenocarcinoma (PDAC), immune oncology (IO) regimens have yet to demonstrate durable efficacy. These poor IO outcomes have occurred even though pancreatic cancer cells exhibit higher levels of immune checkpoint expression than other cancer cell types for which immunotherapy efficacy has been well established, as demonstrated by the characterization of preclinical cell lines (3).

We theorized that the activation of escape or alternate signaling pathways may facilitate resistance to IO agents in PDAC patients. Our group has examined immune checkpoints and ligand-activated alternate signaling pathways in PDAC, demonstrating programmed cell death protein 1 (PD-1)-mediated activation of the proto-oncogene MET in PDAC models *in vitro* and *in vivo*. We observed effective cytotoxicity with the combination therapy of pembrolizumab (an anti-PD-1 monoclonal antibody, mAb) and cabozantinib (a tyrosine kinase inhibitor that also targets anti-MET) (4). These findings formed the basis of an investigator-initiated, phase II clinical trial (NCT05052723) for patients with treatment-refractory metastatic PDAC with a single-arm treatment protocol of pembrolizumab (200 mg, Merck) IV every 3 weeks and cabozantinib (40 mg, Exelixis) PO daily for a cycle length of three weeks. Response and progression were evaluated via the revised Response Evaluation Criteria in Solid Tumors (RECIST v1.1) and iRECIST guidelines. Evaluations were performed every 8 weeks during the first 6 months, followed by every 12 weeks until disease progression or death. Dose modification occurred as previously specified for individual adverse events. Independent dose modification for adverse events that could be clearly attributed to either agent was permitted. Here, we report the case of a patient with metastatic PDAC who had been exposed to two prior lines of systemic therapy and had a durable response to the therapy and remained on the trial for over 2 years before demonstrating radiographic signs of disease progression.

## Case description

**Figure 1:**
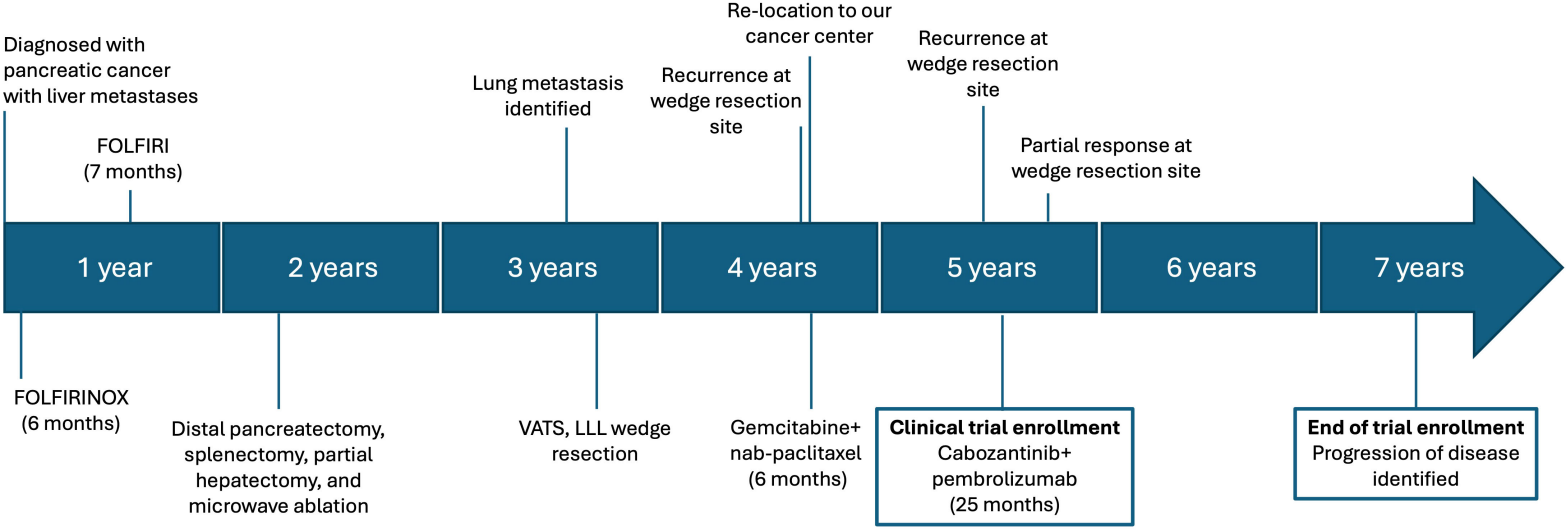
Timeline of diagnosis, treatment, and treatment responses. 5-fluorouracil, folinic acid, oxaliplatin, and irinotecan; FOLFIRINOX. 5-fluorouracil, folinic acid, and oxaliplatin; FOLFIRI. Video-assisted thoracoscopic surgery, VATS. Left lower lobe, LLL.

A 65-year-old female patient was initially diagnosed with PDAC at an outside institution approximately four years before trial enrollment (**Figure 1**). At the time of initial diagnosis, she was taking isoniazid to treat tuberculosis. She developed elevated liver enzymes, which were initially attributed to her isoniazid therapy. She was otherwise asymptomatic and had no abnormal findings on physical examination. Later, radiographic imaging revealed a 12 cm distal pancreatic mass and two liver metastases measuring 11 mm and 18 mm, respectively. A diagnostic biopsy of the primary pancreatic tumor revealed a poorly differentiated adenocarcinoma with features of undifferentiated carcinoma with osteoclast-like giant cells (UCOGC). Next-generation sequencing (NGS, CARIS) of the primary tumor revealed pathogenic *KRAS* G12R and *RNF43* R145X mutations. Additionally, the tumor was PD-L1 negative, microsatellite stable with proficient mismatch repair (MMR), and had an intermediate tumor mutational burden. Since there was no actionable genetic alteration, the decision was made to proceed with the standard of care FOLFIRINOX (5-fluorouracil, folinic acid, oxaliplatin, and irinotecan). Oxaliplatin was discontinued after six months secondary to neuropathy, and the patient completed 23 cycles of FOLFIRI with an excellent radiographic response of the liver metastases. Thus, 1½ years after the initial diagnosis, the decision was made to proceed with a distal pancreatectomy and a partial hepatectomy. Final pathology demonstrated rare foci of residual adenocarcinoma in a background of extensive necrosis in the pancreas, and no tumor was identified in the two partial hepatectomy specimens.

While under surveillance, positron emission tomography/computed tomography (PET/CT) at 13 months after surgery identified a 0.9 x 0.8 cm left lower lobe (LLL) nodule. Two months later, PET/CT demonstrated an increased size of the LLL nodule to 1.2 x 1.1 cm. The increasing size and metabolic activity were concerning for metastatic disease; therefore, video-assisted thoracoscopic surgery with LLL wedge resection was performed, with pathology showing adenocarcinoma most compatible with metastasis from the pancreatic primary tumor. This was reported to be morphologically similar to the original specimen. The patient elected not to receive adjuvant therapy, and 11 months after wedge resection, the patient relocated to Kentucky. A restaging PET/CT scan revealed bilateral pulmonary nodules and increased metabolic activity at the staple line from the prior pulmonary wedge resection, at which point the patient was referred to our cancer center.

**Figure 2:**
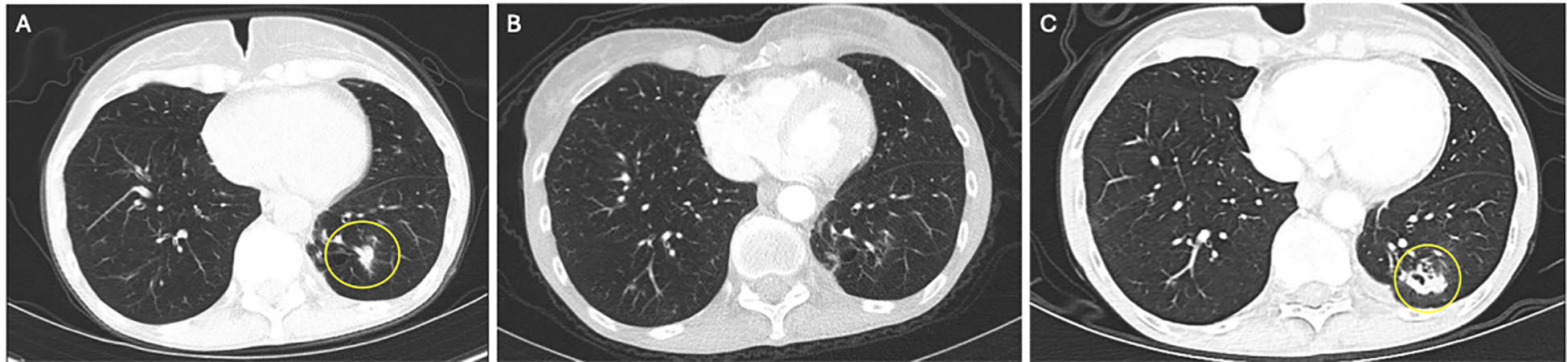
Radiographic imaging of lung metastases. **(A)** Left lower lobe metastasis at the site of a prior wedge resection before clinical trial enrollment. **(B)** R Resolution of the left lower lobe nodule two months after clinical trial enrollment. Stable 8mm right upper lobe nodule not pictured. **(C)** 25 months after trial enrollment, increased soft tissue and ground glass attenuation around the left lower lobe, presumed to be bronchiectatic changes and concerning for disease progression. A CT-guided biopsy was positive for metastasis.

We performed hereditary cancer panel testing (Ambry Genetics), which revealed a variant of unknown significance in the RAD51C gene (p.R260P) and a likely benign variant in the APC gene (p.P1467S). No clinically actionable germline genetic alteration was detected. The decision was made to proceed with the standard of care, gemcitabine plus nab-paclitaxel. The patient received six months of treatment, and restaging scans demonstrated resolution of disease in the LLL and no evidence of new metastases. Consequently, the patient decided to stop chemotherapy and monitor for disease progression. At 5 months of surveillance, a CT scan of the chest revealed a new 12 mm nodular, spiculated density at the LLL wedge resection site (**Figure 2**), and a CT-guided core biopsy showed adenocarcinoma compatible with PDAC metastasis. The patient was then referred for clinical trial evaluation, and at the time of enrollment in our clinical trial, her CA 19–9 levels were 165 U/mL, and her liver enzymes were within normal limits.

During the trial, the patient had an excellent treatment response. At 2½ months after starting cabozantinib/pembrolizumab, she had evidence of a radiologic partial response, with the LLL nodule no longer present and a stable 8 mm right upper lobe (RUL) nodule (**Figure 2**). During treatment, the patient experienced dose delays and reductions of both drugs secondary to adverse events. This included one pembrolizumab dose delay due to grade 2 liver injury, which coincided with a cabozantinib dose delay followed by a dose reduction due to fatigue, anorexia, and weight loss. The patient had one dose delay secondary to grade 3 dermatitis, grade 1 hypothyroidism, and grade 1 diarrhea. She also had one dose delay due to recurrent grade 2 dermatitis and grade 3 hypertension. She had one dose delay secondary to diarrhea alone. Pembrolizumab was withheld for the patient’s final cycle due to fatigue and weight loss. She also received oral prednisone to manage immune-mediated adverse events. After trial enrollment, her CA 19–9 levels dropped from 214 U/mL to 56.6 U/mL. At 9 months, she had stable disease according to RECIST v1.1. She remained on the trial for a total of 25 months, receiving 32 cycles of cabozantinib and pembrolizumab. After 25 months, a surveillance CT chest revealed interval enlargement of two RUL lung nodules and increased attenuation in the LLL, which was concerning for disease progression (**Figure 2**). A PET/CT scan revealed increased metabolic activity in these nodules, corroborating concerns of disease progression. A CT-guided biopsy of the LLL nodule was positive for metastatic adenocarcinoma. Of note, the patient did not have any abnormal symptoms or examination findings. She also had stable CA 19–9 levels, and thus they did not appear to correlate with her disease burden. At this time, the patient was taken off the clinical trial, and cytotoxic chemotherapy was resumed. The patient is still alive at the time of this report. Written informed consent was obtained from this patient for treatment and publication of research results.

## Discussion

In this report, we present a noteworthy patient case from our investigator-initiated phase II clinical trial evaluating cabozantinib and pembrolizumab. This case demonstrates the effectiveness of IO drugs in the setting of metastatic PDAC despite having proficient MMR and prior exposure to two lines of therapy. This patient was enrolled in our trial for 25 months before disease progression was identified, which is remarkable considering that the historical median progression-free survival in the refractory setting is 2 months (5). Notably, the pathology demonstrated UCOGC, which is an established variant of PDAC (6). Its distinctive feature is the presence of osteoclast-like giant cells, which resemble those of a giant-cell tumor of the bone (7). In tumor specimens, UCOGC has been associated with conventional PDAC, mucinous cystic neoplasms, and intraductal papillary mucinous neoplasms, and it is found in less than 1.4% of pancreatic cancer cases (7, 8). Immunohistochemical features have been found to include expression of CD68, PD-1, and PD-L1; a low Ki-67 proliferation index; and negative AE1/AE3 and p53 expression (8–10). The reported survival for patients with UCOGC is highly variable, ranging from 12 months to several years (8, 11–13). Little data are available on the responsiveness of UCOGC to treatment; however, some case reports have demonstrated the limited effectiveness of chemotherapy (14). Here, we demonstrate an effective and durable treatment response of UCOGC to IO drugs, leading to prolonged survival despite having metastatic disease.

There are potential mechanisms for improved survival with IO drugs in UCOGC. The first mechanism may occur through therapeutic targeting of the PD-1/PD-L1 axis. Luchini et al. evaluated PD-1 and PD-L1 expression in UCOGC and observed PD-1 expression (albeit limited) in 44% of cases and PD-L1 expression in 63% of cases, both of which are higher than PD-1/PD-L1 expression levels in non-UCOGC PDACs (9). Second, another report on UCOGC showed a greater frequency of PD-L1 expression concomitant with increased tumor-infiltrating lymphocytes (10). Both articles support the use of an IO drug approach to manage UCOGC PDACs. Apart from the report on our clinical trial patient, two other case reports further highlight the efficacy of IO drug therapy (*i.e.*, pembrolizumab) for metastatic UCOGC of the pancreas (15, 16). In one case, tumor sequencing revealed a high tumor mutation burden. Following radiation therapy to the primary tumor and metastases, the patient was started on pembrolizumab as a third-line therapy and had a partial response to treatment of the primary tumor and lung metastasis. In another case, a patient initially diagnosed with non-small cell lung cancer with abdominal lymph node metastasis was found to have PD-L1 expression and was started on pembrolizumab. This therapy led to a response in the lung metastases, but not in the primary tumor, which was later diagnosed as pancreatic UCOGC. In our clinical trial, we also incorporated cabozantinib in combination with pembrolizumab. Cabozantinib appears to further enhance cytotoxic immune responses, since its activity lies in the inhibition of tyrosine kinases, some of which are involved in immune cell regulation. Indeed, cabozantinib has been shown to be effective in killing cancer cells when combined with immune-checkpoint inhibition (17). It should be noted that functional translational studies were not conducted to evaluate the impact of these IO targets on treatment response. Additional cell-surface targets or members of related immunomodulatory pathways should be investigated to further refine synergistic treatment strategies for UCOGC and other PDAC variants.

In summary, our patient’s case demonstrates prolonged survival after exposure to multiple lines of therapy, highlighting the importance of personalized medicine and translational research in driving the development of novel therapeutic regimens. Given the historically poor response to immune checkpoint inhibitors, our synergistic approach of targeting both PD-1 and MET shows great promise for select patients with metastatic PDAC, particularly for those with the UCOGC variant. Since osteoclast-like giant cells have been detected in many other cancer histologies, including breast, liver, and lung cancers (18–20), and considering the prolonged response to cabozantinib and pembrolizumab in our UCOGC patient, this combination therapy could be considered for patients with osteoclast-like giant cells of any cancer type.

However, inherent limitations exist for a report of a single patient with a rare disease variant. The unique biology and treatment history of this single patient may prevent the generalizability of treatment efficacy. Additionally, UCOGC components were not identified in metastatic sites based on the available material. Not only translational correlative studies but also randomized prospective studies are imperative to assess the potential for efficacy of pembrolizumab and cabozantinib in all patients with UCOGC and other variants of PDAC.

## Data Availability

The raw data supporting the conclusions of this article will be made available by the authors, without undue reservation.
